# A Program for Weaning Children from Enteral Feeding in a General Pediatric Unit: How, for Whom, and with What Results?

**DOI:** 10.3389/fped.2018.00010

**Published:** 2018-01-25

**Authors:** Justine Mirete, Béatrice Thouvenin, Gaelle Malecot, Morgane Le-Gouëz, Christel Chalouhi, Catherine du Fraysseix, Aurélie Royer, Anais Leon, Clément Vachey, Véronique Abadie

**Affiliations:** ^1^General Pediatrics Department, Necker University Hospital, Assistance Publique-Hôpitaux de Paris, Paris, France; ^2^Paris Descartes University, Paris, France; ^3^Clinical Investigation Center, INSERM CIC 1431, University Hospital of Besançon, Besançon, France

**Keywords:** enteral feeding, tube dependency, weaning, gastrostomy, eating disorders, feeding difficulties

## Abstract

**Objective:**

To describe a series of children who were hospitalized for a tube-weaning program in the general pediatric ward of a pediatric tertiary university hospital: describe our method, to determine the success rate of our inpatient pediatric tube weaning program, and search for relevant factors linked to its success or failure.

**Method:**

We analyzed the medical files of consecutive children who were hospitalized for gastric-tube weaning over an 8-year period. We analyzed outcomes in terms of feeding and growth with at least 2 years of data. Success (weaning within 3 months) and failure were compared by characteristics of children.

**Results:**

We included 37 children (29 females) with mean (SD) age 31.4 (21) months. Most had a severe medical history (30% prematurity; 50% intrauterine growth restriction, 50% neurological and genetic anomalies). The weaning program was successful for half of the children. Factors linked to success of the program were female sex (*p* = 0.0188), normal neurodevelopment (*p* = 0.0016), nasogastric tube (*p* = 0.0098), and with <24 months on EF before the stay (*p* = 0.0309).

**Discussion:**

Comparing the efficiency of various methods and results among teams was difficult, which indicates the need to establish consensus about the outcome criteria. We confirm the need for these types of stays and programs.

## Introduction

The benefits of nutrition by a gastric tube in preterm infants unable to adequately feed without assistance have been known since the end of the 19th century (1). The first studies in the 1950s showed a positive impact of enteral feeding (EF) by nasogastric tube (NGT) on survival and growth in children (2), and the advantages of gastrostomy were reported in the 1960s (3). The number of children benefiting from EF by NGT or gastrostomy is increasing with a concomitant decrease in the mean age of infants treated, which is mainly due to advances in prenatal diagnoses and medico-surgical neonatal resuscitation (4).

Although these strategies brought considerable benefits to children, they are also associated with some deleterious side effects, particularly in terms of feeding and eating disorders. In fact, some children, despite successful treatment or improvement in medical condition, seem incapable of resuming normal oral feeding. These feeding and eating disorders, in particular avoidant/restrictive intake disorders, lead to tube dependency. They are associated with previous underlying physical causes and/or current functional and psycho-emotional causes. They are characterized, with variable levels of intensity, by an obvious disinterest in food, an attitude of opposition or avoidance to food, an aversion to food in general or at least all foods with the exception of one or two, a phobia of introducing food into the mouth or food of certain textures, a prominent pharyngeal reflex as well as effortless vomiting, and a hypersensitivity affecting the whole body. These features often lead to significant psychological problems affecting intrafamilial and social relationships (5, 6).

In most cases, the care of these children during weaning can be provided as ambulatory care by the pediatric teams who previously treated the child, by progressively decreasing EF to lead to normal oral feeding. A multidisciplinary re-education approach, such as that provided by specialized ambulatory structures for children with development or psychological difficulties, is often required. Nevertheless, some children remain resistant to the care provided in the outpatient setting and require a full-time stay for more intensive support. Many specialized clinics exist worldwide, offering stays to assist weaning from tube feeding, the best known being in Europe, in Graz in Austria. Some specialized units also operate within pediatric hospitals, such as the Feeding and Swallowing Centre at the Children’s Hospital, WI, USA (7–12). In France, some structures providing post-acute and rehabilitation care recently started offering weaning stays, but there are few such service providers and limited available data regarding their methods and results. In public hospitals for children, general pediatric, pediatric gastroenterology, or child psychiatry wards sometimes provide care for children in this situation, but due to the functional structure of public hospitals in France, predominantly dedicated to short-term stays and acute diseases, the lack of medical team expertise for treatment has not led to well-structured strategies.

The general pediatric department of Necker Hospital in Paris encompasses a reference center for rare diseases dedicated to “Pierre Robin sequences and congenital disorders of sucking and swallowing.” For the past 15 years, it has offered children resistant to EF weaning access to an expert and streamlined in-patient program managed by a specialized multidisciplinary team.

The objectives of this study were to describe a series of children who were hospitalized for a tube-weaning program in the general pediatric ward of a pediatric tertiary university hospital, to describe our method, to analyze the results of our program with at least 2 years of follow-up, and search for relevant factors linked to its success or failure.

## Materials and Methods

### Patients

We selected all children hospitalized from 2008 to 2015 (which coincided with the implementation of the electronic health records system at Necker Hospital and launch of our specific reference center database) for EF weaning. The decision to hospitalize these children had been medically decided by one of the two main pediatricians of the ward, a few weeks beforehand if the tube weaning had failed or was resisted in ambulatory care after at least 1 year of attempt. These children were fed by NGT or gastrostomy. All children hospitalized for weaning stays were included in the study regardless of age, length of EF and initial pathology as long as they had safe swallowing abilities and a stable cardiopulmonary status. We excluded children whose stay was less than 5 days and those who died during the study.

### Program

Each child benefited from a program of multidisciplinary management, which included sessions with physicians, nurses, a psychomotor (occupational) therapist, a speech-language pathologist, psychologists and dietitians, and was applied in the following steps:

– Initial time set aside for observation and evaluation of causes for the resistance.
– First appraisal by the multidisciplinary team.
– Preparation of a therapeutic contract with parents, initially for 1 week and renewable a maximum of two additional times, depending on the child’s progress.
– Reduction of EF during the first 3 days according to the child’s capacity:
  – “Safe swallowing” when drinking water but eats nothing: restriction of fluids but not calories, transition to four feeds of high-energy foods contained in the smallest possible volume (1.5 kcal/ml).
  – “Safe swallowing” while eating a few spoonful of food and drinks water: fluid and calories restricted by approximately 25% while maintaining four feeds.
  – Able to eat the equivalent of 1 yogurt or 150 g of mixed foods orally per day: 50% withdrawal of EF while completing only two of the four meals by gastric tube.

During the stay, we reduced the EF meal by meal, stopping EF when oral intakes reach about 2/3 of the prescribed volume.

– Therapeutic principles and timetable. We endeavor to create a non-clinical atmosphere. The child shares a “mother–child” room with one parent, while the other parent is able to stay in the hospital family housing facility, if both parents choose to be present at Necker. Assessments including examinations and blood tests are performed before admission to best gain the child’s confidence and avoid stress.

The timetable, displayed in the child’ room, consists of:

– daily medical meetings,
– once or twice a week with the senior referring physician,
– multiple times a week with the psychologist,
– daily sessions with the psychomotor therapist alone or with the psychologist or speech-language pathologist,
– once or twice-weekly individual sessions with the speech-language pathologist,
– regular meetings with the dietitian.

Mealtimes are for the most part publicized; that is, they were taken in a room with an expert professional. Morning and evening meals can be taken with the parents and are more or less supervised by the nurse of the general pediatric ward assigned to the child. The dietitian meets with parents at the beginning of hospitalization to adapt meal trays, as best possible. Meal trays, known as “discovery,” are offered with recreational foods that are appreciated by children. Parents are allowed to bring in additional foods. In this case, the dietitian records what is ingested, calculates ingested calories, and adjusts subsequent meal trays accordingly.

The remaining time is free and can be spent with the early childhood educator in the unit’s playroom or in the classroom with the teacher, depending on the child’s age and neurodevelopmental capacities. Free time spent outdoors is also possible. Weekends are periods of rest, during which return visits to the family home are authorized as long as they are not likely to destabilize the child or alter the burgeoning relationship with the medical team.

Principles of management are based on a wide-ranging supportive, flexible policy but follow a relatively intense rhythm, which is reassuring on a medical level. The parents are free (when possible) from worries relating to risks associated with the withdrawal of nutrition, particularly with regard to weight loss and risk of deprivation. They are supported psychologically, heard by various team members, always ensuring that no guilt is assigned. Team members and parents both engage in the day-to-day difficulties encountered with the child, especially in the context of nutrition, but also in terms of education and behavior. Time set aside for medical observation also allows for reflection on the diagnosis, previous history, positive outcomes, and other elements, which help clarify the current situation and often lighten the load. The care has a psychomotor therapeutic approach, which includes activities that do not exclusively focus on nutrition but aim to develop multi-sensorial sensitivity and balanced emotional regulation. Particular attention is attributed to fostering interactive competences of the child, especially strengthening the parent–child bond as well as the family dynamic. Everything is focused on placing the child and the parents in an atmosphere of trust and consideration with regard to progress.

### Patient Analysis

To describe the cohort, the following data were collected from hardcopy records or computer files for each hospitalized child:

– Personal data: age, gender, gestational age, existence of intrauterine growth restriction, profession, and socio-occupational categories of the parents according to nomenclature of the National Institute of Statistics and Economic Studies. We condensed the six categories into three groups:
  ○ Categories 1 and 2: agriculturalists, artisans, retailers, business managers.
  ○ Categories 3 and 4: executives, university educated professionals, middle management and “intermediary” occupations.
  ○ Categories 5 and 6: employees and manual workforce.
– Predominant diagnosis: the “predominant diagnosis” was most pertinent physical diagnosis that justified the EF.
– Psychomotor or cognitive development: psychomotor development had been clinically evaluated during hospitalization while attempting weaning from EF or at a later stage during follow-up consultations. This clinical evaluation allowed for classifying children into two distinct groups, those with normal or suboptimal development and those with a deficiency.
– Feeding history: age at first appearance of difficulties, age at EF implementation, type of nutritional support (NGT or gastrostomy), length and tolerance of treatment, age at admission, length of hospital stay, and quantification of EF supplementation at admission.
– Anthropometric measures: the weight, length, BMI (body mass index) of each child were measured with the Sempé and Pédron’s curves on the day of admission, every day during the stay, on the discharge day, and at each follow-up visit. We expressed them in mean, median, percentile, and *z*-scores.

We then analyzed the success or failure of tube weaning. Tube weaning was scored as “success” when it occurred during hospitalization or when it was unambiguously initiated and dynamically perpetuated and led to a complete and definite cessation of NGT or gastrostomy use within 3 months of discharge. Tube weaning was scored as “failure” for children weaned at a later stage after discharge (>3 months) or who did not wean during the timeframe of the study. The 3-months’ postdischarge time was arbitrarily defined because it often reflected an effective outcome.

In children with successful weaning, the consequences of weaning on growth were studied. During follow-up consultations, we sought to emphasize changes to growth curve trajectories in terms of height and weight measurements taken at admission.

### Statistical Analysis

We sought to identify factors associated with weaning “success” or “failure” of the hospital stay by comparing children’s medical records and personal information in both “success” and “failure” groups. Since most of quantitative variables had a not normal distribution, data were described as median and inter quartile ranges (IQR 25–75). Qualitative data were described as number and proportions. Chi-square and Fisher exact tests were used to compare qualitative variables. Student’s *t* test or the non-parametric Wilcoxon test was used to compare quantitative variables. Analysis involved use of SAS 9.4 (SAS Institute Inc., Cary, NC, USA). All tests were two-sided and *p-*values <0.05 were considered statistically significant.

## Results

### Characteristics of the Cohort

Between January 2008 and December 2015, 40 children were admitted to the general pediatric ward for an EF weaning stay. Three were excluded: the first died in the year following the stay due to an infectious event unrelated to the eating problem, the second stayed in the hospital for only 2 days because the parents were not satisfied with the program, and the third had weaned between the last consultation to prepare for the stay and the day of admission.

The study thus included 37 children (29 girls, male–female ratio 3.6) (Table 1). Overall, 30% of children were preterm births (<37 weeks’ gestation), 8% extremely premature (<28 WG), and 49% showing intrauterine growth restriction. In all, 62% had parents of socioeconomic categories 5 and 6, considered disadvantaged, which was higher than in the French general population (48% in 2015, https://Insee.fr website). In contrast, categories 3 and 4, considered privileged, were under-represented, which is unexpected for a hospital in central Paris.

**Table 1:** Characteristics of the cohort.

Characteristics	*n* = 37 (%)
Male	8 (22)
Female	29 (78)
Birth term (WA)	
<28	3 (8)
(28–32)	2 (5)
(32–37)	5 (14)
Full term birth	27 (73)
IUGR	18 (49)
PSOC 1 and 2	3 (8)
PSOC 3 and 4	11 (30)
PSOC 5 and 6	23 (62)

*WA, weeks of amenorrhea; IUGR, intrauterine growth restriction; PSOC, professions and socio-occupational categories of parents*.

### Predominant Diagnosis

The predominant diagnoses greatly differed among children, ranging from significant neurological pathologies to complete absence of underlying physical pathologies, neither criteria for exclusion. Half of the children had a neurological or genetic anomaly and only a few [*n* = 4 (11%)] had an isolated psychogenic eating disorder (Table 2).

**Table 2:** Predominant diagnoses of children.

Diagnoses	*n* = 37
Genetic syndromes	13 (35%)
Pierre Robin	4
Williams	1
Noonan + chylothorax	1
Netterthon	1
VACTERL	1
Malformative syndrome, chromosomal anomaly	5
Neurological impairments	6 (16%)
NBD with moderate mental deficiency	4
Isolated NBD	1
Brain malformation	1
Prematurity <32 WA	5 (14%)
Isolated congenital heart disease	3 (8%)
GER or other digestive problems associated with a psychological component	3 (8%)
Post-traumatic feeding disorder (prematurity, transplant, volvulus)	3 (8%)
Infantile anorexia with disturbed mother–child bond	4 (11)

*NBD, neonatal brainstem dysfunction; GER, gastroesophageal reflux; VACTERL, vertebral defects, anal atresia, cardiac defects, tracheoesophageal fistula, renal anomalies, and limb abnormalities; WA, weeks of amenorrhea*.

### Psychomotor or Cognitive Development

Fifteen children (40.5%) were considered deficient in cognitive development and 22 (59.5%) as having normal or suboptimal development.

### Eating History

For the 37 children, age at which eating difficulties became apparent was precocious (median = 0.2 months, IQR = 0.2–3 m), so, most presented a neonatal pathology with an age of EF implementation of 1 month (median, IQR = 0.2–6 m). In all, 23 children (62%) had gastrostomy, 17 (74%) associated with Nissen type anti-reflux surgery; the remaining children were fed by NGT. Thirteen (35%) children had problems tolerating EF, especially because of frequent vomiting; 9 children underwent gastrostomy, 7 of these associated with Nissen surgery, and 4 with NGT. The median (IQR) duration of EF was 29 (13–52) months.

The median (IQR) age at hospitalization was 26 (16–46) months. On arrival at the service, 23 children (62%) received >90% supplementation by EF and 13 (35%) between 50 and 90% supplementation. None of the children ate half or more than half of their ration orally. The median (IQR) time of hospitalization was 11 (8–19) days.

### Weaning Success Rate

For 19 children (51%), the stay was successful immediately or shortly thereafter: 11 were weaned during hospitalization and 8 in the following 3 months postdischarge. Another 8 children were weaned in the following 12 months, by staying at the “Centre des Côtes” (post-acute care and rehabilitation facility) or by following monitoring consultations. Ten children remained EF-dependent (27%).

### Growth Monitoring

On arrival, the BMI of the whole group was 14.9 (median: 14.8—percentile 25), *z*-score = −0.18. At discharge, the BMI was 14.5 (median 14.5—percentile 25), *z*-score = −0.27. The median (IQR) weight loss during hospitalization was 2.7% (0–4.7%). Among the 27 weaned children, the mean follow-up of growth monitoring was 27.7 months (median: 22.4 months). At this term, their BMI was 14 (median 13.9—percentile 20, *z*-scores = −0.26). Twenty-three children (85%) followed their initial percentile height and weight growth trajectories. One child dropped to a lower percentile growth curve in height, but he had severe scoliosis, which greatly affected his standing height measurement. One other child dropped to the lower percentile growth curve both in height and weight because of incorrect food intake. Two children were lost to follow-up.

### Comparison of “Success” and “Failure”

Success (= definitive tube weaning at discharge or within 3 months of discharge) was more frequent than failure (= weaning >3 months after discharge or no weaning at the end of the study) for girls (*p* = 0.0188) and for children with normal or limited cognitive development (*p* = 0.0016), with NGT (*p* = 0.0098), and with <24 months on EF before the stay (*p* = 0.0309). It was also more frequent with longer stay in the program (*p* = 0.02) (Table 3).

**Table 3:** Characteristics associated with weaning outcomes during hospitalization (univariate analysis).

Characteristics	Success *n* = 19	Failure *n* = 18	*p*
Number of females	18 (95)	11 (61)	**0.0188**
Gestational age, median (IQR)	38 (36–39)	36.3 (4.4)	0.46
IUGR, *n* (%)	8 (42)	10 (56)	0.52
PSOC *n* (%)			0.8
1 and 2	1 (5)	2 (11)	
3 and 4	6 (32)	5 (28)	
5 and 6	12 (63)	11 (61)	
Predominant diagnosis, *n* (%)			0.56
Genetic syndrome + neurological impairments	9 (48)	11 (61)	
Cardiovascular disease, prematurity <32 WA posttraumatic ED	5 (26)	5 (28)	
Digestive problems associated with a psychological component	5 (26)	2 (11)	
Level of development, *n* (%)			**0.0016**
Normal or limited	16 (84)	6 (33)	
Deficient	3 (16)	12 (67)	
Age at start of feeding difficulties, months, median (IQR)	0.7 (0.2–3.5)	0.2 (0.2–3)	0.62
Age at start of enteral feeding (EF), months, median (IQR)	2 (0.2–7)	0.75 (0.2–6)	0.9
Type of tube feeding, *n* (%)			**0.0098**
Gastrostomy	8 (40)	15 (83)	
Nasogastric tube	11 (60)	3 (17)	
Length of EF, months, median (IQR)	15 (6–52)	44.5 (23–59)	0.13
<24 months, *n* (%)	12 (63)	5 (28)	**0.0309**
≥24 months *n* (%)	7 (37)	13 (72)	
Tolerance to EF, *n* (%)			0.64
Good	13 (68)	11 (61)	
Bad	6 (32)	7 (39)	
Age at hospitalization, months, median (IQR)	22 (8–40)	31 (19–49)	0.24
Length of stay in the program, days, median (IQR)	15 (10–26)	10 (8–11)	**0.02**
Quantity of EF intakes before weaning stay, *n* (%)			0.61
≥90%	13 (68)	11 (64)	
50–90%	6 (32)	7 (38)	

## Discussion

Here, we describe a series of children who were hospitalized for a tube-weaning program in the general pediatric ward of a pediatric tertiary university hospital. Hospital stays for EF weaning were beneficial because during a short stay of 2–3 weeks, half of the children showed rapid weaning and three quarters definitive weaning within a year after the stay, without decline of growth trajectory. Success was more frequent than failure for girls and for children with normal or limited psychomotor development, NGT, less EF time before the stay and more time in the program.

Difficulties encountered in weaning from EF due to oral eating issues are a major problem for some children and their families, even when the predominant underlying cause requiring implementation of nutritional support is resolved. The frequency of cases resistant to weaning in the ambulatory care setting is difficult to evaluate, because it depends on the tolerance of families to the continuation of EF, processes available in ambulatory care to achieve weaning, and the team’s awareness of the existence of these structures. The fact that in 8 years, 40 such children were admitted to a general pediatric expert ward confirms the demand for interventions, which requires increasing these weaning stays in expert centers throughout France when management in ambulatory care is failing.

For the most part, children in this series were born premature or with intrauterine growth restriction. Logically, these children are more difficult to wean in ambulatory care because parents and doctors particularly emphasize weight gain in this neonatal period with the objective to compensate for impediments to stature and weight growth, which may impose a more prolonged and intense period of EF. Moreover, such situations are often stressful to parents, and also, premature children are for the most part more vulnerable than term infants.

Families in this series were predominantly from disadvantaged socio-occupational categories. Their likely weak financial means cannot be the cause of the ambulatory care failure because in France, medical care is free for all children with severe chronic disease, especially those with home EF. Our children were often at the center of an untreated psychopathological family dynamic, which may explain the failure of ambulatory care and the benefits of a longer stay in centers for post-acute care and rehabilitation. The scope of this study did not allow for objective evaluation of the contribution of maternal psychopathology or the dysfunction of the mother–child relationship. This remains to be examined in future prospective studies, which should additionally evaluate previous maternal history of eating disorders, both with standardized scales.

The predominant diagnoses of children in our series were very heterogeneous. Nonetheless, only a few exclusively presented an eating problem of psychogenic origin. This finding can be explained by EF weaning in ambulatory care being less difficult for these children. Moreover, that most children presented a severe organic pathology shows that EF is not used excessively in the long term and indications for its use are initially fully justified. As a whole, the epidemiological description of our series showed rather severe conditions, which is explained by these children being the more resistant among the tube-dependent children of similar age (13).

Weaning stays offered in international clinics or specialized units use programs closely related to ours with a multidisciplinary approach (14, 15). In a recent systematic review, Sharp et al. described eight studies describing inpatient stay, only two having similar multidisciplinary team of caregivers as ours (16). In fact, interventions by a psychologist, an occupational therapist, a physiotherapist, a dietician, and a speech-language pathologist, are useful in these complex situations. Our method of decreasing EF supplementation at the beginning of the stay is more progressive than other methods, with the decrease being 50% at the second to third day from admission in the Graz model. The intensity of management in terms of number of daily sessions is perhaps lower in a pediatric service than in a dedicated unit, in which all staff members are exclusively dedicated to weaning stays.

Our results show a short-term success rate in 51% of children admitted. This is lower than the rate reported by other studies. Indeed, Shalem et al. reported a success rate of 86% in Israel, with 24 of 28 children weaned (12). Hartdorff et al. also reported an 86% success rate in Amsterdam, with 18 of 21 children weaned (11). In the United Kingdom, Wright et al. reported a success rate of 78% but with a longer follow-up (8). Finally, Trabi et al. found a greater success rate in Graz, 92%, with 203 of 221 children weaned (7). Success rates between teams are not easily comparable. First, the definition of successful weaning itself is not identical among centers, in terms of the practical details of feeding regimes adopted once weaned from EF and also the timeframe considered between leaving the facility and weaning. We defined successful weaning as the discontinuation of NGT or gastrostomy but with the concomitant absence of a slowing growth rate and a rapid resumption of an ascending and regular weight gain rate as well as a return to a normal social lifestyle. These stipulations are not comparable between specialist centers or are poorly described in publications. Our pediatricians’ insistence on growth, within a pediatric ward in a tertiary hospital for children, may be more stringent than that of a child psychiatry unit team. Moreover, weaning should be followed by a resumption of eating in a harmonious manner that is more or less balanced and without direct conflict. It is not about weaning “at any cost,” the child must succeed in eating various foods with different textures and with a significant reduction in aversion to foods. Then again, what exactly is meant by “harmonious eating” is subjective and thus varies among teams and families and is poorly evaluated along the way. Therefore, this definition precludes a comparison of different methods and structures, especially those lacking a medium- to long-term follow-up. The pathologies in our series were for the most part severe in children with often compromised developmental level. Our recruitment is not comparable to that of some specialized clinics, which can in part explain the lower success rate of our weaning stays. Finally, the lack of funding for public hospitals and the inconvenience, in the general pediatric ward, of a team often solicited in emergencies and other related care as well as the suspension of weaning stays during the infectious winter season may help explain our results. This hypothesis justifies the development of weaning stays in structures equipped for medium-term stays, in a less clinical setting, and one better adapted to providing re-education/rehabilitation type care as compared with services offered in a university hospital.

The consequences of weaning from EF on height and weight growth of children in this series are satisfactory because only one child dropped to a lower percentile growth curve in weight and height in the mid-term (2 years). Our data are comparable to the Wilken et al. study showing rapid weaning with no impact on *z*-scores, namely 2 SD from the mean (17), and to Wright et al. who reported a decrease in BMI at weaning in the absence of a concomitant decrease in height (8). However, the other studies did not present post-weaning growth data, although these are an important indicator of the quality of patient management.

We sought to identify factors that could predict weaning success or failure of our program. Because of the small sample, the analysis of our results requires caution. Factors associated with failure were male sex and reduced level of psychomotor or cognitive development. We do not have an explanation for the association with gender. In contrast, delays in development no doubt reduce a child’s capacity to process what is being presented, which may indicate that these children would benefit from a more realistic dialog, with the setting of firmer strategies to prevent problems associated with oral feeding. EF by gastrostomy and EF of more than 24 months before weaning were also negative factors. These two factors are interconnected. Children with gastrostomy are in fact those with more serious underlying pathologies and require an extended period of nutritional support. Indeed, gastrostomy is more comfortable than NGT for children and frees the mouth for oral eating, but for children and their families, it implies longer term treatment, with weaning seemingly less urgent than with a NGT. Finally, a longer period of hospitalization seems to predict success but is biased on the fact that children for whom our treatment was not so effective during the first week left more rapidly than those for whom the third week of stay was important for success.

## Conclusion

We offer children resistant to weaning from gastric tube feeding in ambulatory care an efficient program consisting of a short stay with multi-professional intensive management in a hospital structure, which ensures their medical safety in the short and medium term. To allow for valid comparisons between different teams, different strategies, and results of such programs, more precise and consensual definitions of the parameters of outcome and oversight are needed, especially long-term follow-up of growth and eating behavior in these children.

## Ethics Statement

We only analyzed files of patients that were treated in our ward. This is in agreement with the policy of ethics of our institution.

## Author Contributions

JM and BT collected the data and participated to the manuscript writing. GM, ML-G, CC, CF, AR, and AL are the pediatricians, the occupational therapist, the speech therapist, and the nurse who were in charge of the children during the stays. They all participated to the study design and corrected the manuscript. CV made the statistical analysis. VA is the head of the service, designed the study, and wrote the manuscript. All the authors validated the final version.

## Conflict of Interest Statement

The authors declare that the research was conducted in the absence of any commercial or financial relationships that could be construed as a potential conflict of interest.
